# Nosocomial Bacterial Bronchopneumonia and SARS-CoV-2 Pneumonia in Patients with Traumatic Injuries: Imaging Aspects and Macroscopic and Microscopic Findings of Lung Tissue

**DOI:** 10.3390/diagnostics14232737

**Published:** 2024-12-05

**Authors:** Georgiana-Denisa Gavriliţă, Ştefania Ungureanu, Ecaterina Dăescu, Mircea-Nicu Gavriliță, Cristian-Cosmin Ţîncu, Alexandra Enache

**Affiliations:** 1 Doctoral School, Victor Babes University of Medicine and Pharmacy Timisoara, 300041 Timisoara, Romania; denisa.tincu@umft.ro; 2 Institute of Forensic Medicine Timisoara, 300041 Timisoara, Romania; daescu.ecaterina@umft.ro (E.D.); gavrilitamircea@yahoo.com (M.-N.G.); enache.alexandra@umft.ro (A.E.); 3 Ethics and Human Identification Research Center, Department of Neuroscience, Discipline of Forensic Medicine, Bioethics, Deontology and Medical Law, Victor Babes University of Medicine and Pharmacy Timisoara, 300041 Timisoara, Romania; 4 Discipline of Forensic Medicine, Bioethics, Deontology and Medical Law, Department of Neuroscience, Victor Babes University of Medicine and Pharmacy Timisoara, 300041 Timisoara, Romania; 5 Department I of Anatomy and Embryology, “Victor Babes” University of Medicine and Pharmacy Timișoara, 300041 Timisoara, Romania; 6 “Pius Brânzeu” County Emergency Clinical Hospital, 300723 Timisoara, Romania; cristiancosmintincu@gmail.com

**Keywords:** SARS-CoV-2 pneumonia, nosocomial bronchopneumonia, traumatic injuries

## Abstract

Background: Patients with traumatic injuries often represent the best hosts for healthcare-associated infections, especially pneumonia or bronchopneumonia. The severe acute respiratory syndrome coronavirus 2 (SARS-CoV-2) pandemic raised serious problems in the diagnosis and treatment of patients that had a SARS-CoV-2 infection and associated nosocomial bacterial bronchopneumonia. In forensic medicine, these aspects need to be considered when establishing the cause of death and the distinction between the two types of bronchopneumonia is of particular importance. Methods: We present nine cases that were autopsied at the Institute of Forensic Medicine Timisoara between 1 June 2020 and 31 December 2021, that presented traumatic injuries, a SARS-CoV-2 infection, and bronchopneumonia. Results: We focused on the main findings of the macroscopic and microscopic aspects of lung tissues. Conclusions: We consider that the aspects we highlighted in this study, can be very useful in forensic practice in cases with a pluri-factorial pathology.

## 1. Introduction

When the body is attacked by a traumatic agent, in addition to a local response, a series of general, morpho-functional changes occur, that cause disorders of biochemical and endocrine-humoral constants. These are then expressed in a clinical symptomatology which can either be specific to a certain type of trigger or represented by a vague and general response. This whole set of pathological manifestations is expressed synthetically using the term of traumatic disease. Therefore, the severity of a traumatic disease depends on multiple factors which include the body’s reactivity to traumatic agents, a reactivity that can also be influenced by the presence of pre-existing pathological conditions.

Despite a correct course of treatment, the potential infectious complications during hospitalization can add to the effect of the traumatic agent. In addition, the role of the pre-existing pathological disease can often affect the patient’s evolution in a negative manner. These aspects need to be considered when establishing the cause of death, regarding which represents the decisive role in the process of death.

Patients with traumatic injuries often represent the best hosts for healthcare-associated infections, especially pneumonia or bronchopneumonia. This is probably because they are either subjected to many surgical interventions or invasive maneuvers or are immobilized in bed for long periods of time, and most of them already have pre-existing pathological conditions [1].

Since the assessment of respiratory symptoms like cough, chest pain, and dyspnea is not always appropriate, particularly in situations involving diminished awareness, chest injuries, and lung contusions, diagnosing pneumonia or bronchopneumonia in trauma patients can be challenging [2].

The World Health Organization (WHO) defines a healthcare-associated infection (HAI) as ‘an infection occurring in a patient during the process of care in a hospital or other healthcare facility, which was not present or incubating at the time of admission’. Nosocomial pneumonia, also known as hospital-acquired pneumonia (HAP), is an infectious lung inflammatory condition that does not exist at the time of hospitalization; it was not incubating at the time of admission and develops more than 48 h later. One important subset of HAP is ventilator-associated pneumonia (VAP). More than 48 to 72 h after tracheal intubation, it manifests in individuals who have an artificial airway. VAP accounts for over 80% of pneumonias contracted in the intensive care unit (ICU) and affects 10% to 20% of patients on mechanical ventilation for longer than 48 h [3]. According to the guidelines, the following are necessary for the diagnosis of HAP and VAP: new lung infiltrates on chest imaging, respiratory decline, fever, and productive cough [3].

The SARS-CoV-2 pandemic raised new problems in the diagnosis and treatment of patients that had a SARS-CoV-2 infection and also associated nosocomial bacterial bronchopneumonia.

SARS-CoV-2 may promote bacterial colonization and adhesion to host respiratory tissue in COVID-19 patients admitted to intensive care units (ICUs), resulting in mixed infections. However, a bacterial super-infection could make it easier for the virus to propagate throughout the body, raising the possibility of septic shock [4]. Often, is difficult to appreciate whether these are co-infections or super-infections, especially when specified tests were not performed at the right time. Furthermore, we cannot exclude other respiratory viral infections or fungal infections.

The microorganisms that cause nosocomial pneumonia differ depending on the population being investigated, the length of hospitalization and critical care stay, the diagnostic techniques employed, whether antibiotic therapy has been administered before, and the length of time spent on mechanical ventilation. In the most studies, *Acinetobacter baumannii* was found in 93–95% of cases and is the most frequently isolated germ [5,6], then *Streptococcus pneumoniae*, *Pseudomonas aeruginosa*, and *Staphylococcus aureus*, especially the methicillin-resistant form. Fungi like *Candida albicans* and *Candida tropicalis*, as well as bacteria including *Escherichia coli*, *Enterococcus faecalis* [7], *Enterobacter*, *Haemophilus influenza*, and *Serratia maltophilia*, are also common nosocomial pathogens [8].

Autopsies have always been crucial in providing valued information on the pathological changes that occur in various diseases. This holds true in various clinical medical domains in addition to the forensic domain. Throughout the COVID-19 outbreak, forensic sciences and medico-legal autopsies had a major role in offering precious knowledge, especially on the morpho-pathological changes of the various tissues and organs. Examining the cause of death, medical history, and case reviews are the first and most important steps in forensic autopsy with reference to epidemic prevention in order to successfully confirm a SARS-CoV-2 infection or a suspected case [9]. Few studies have examined the wide range of COVID-19 pneumonia differential diagnoses, whereas the scientific community has concentrated on the clinical and radiological characteristics of COVID-19 pneumonia since the outbreak [10].

When examining autopsy results and determining the cause of death, forensic pathologists should take into account the potential for co-infections and super-infections. Furthermore, there might be instances where a patient who already had a disease or an immunodeficiency had COVID-19 and another infection; this can make interpreting autopsy results much more difficult and necessitate a more thorough investigation. In order to create better treatment plans and stop the virus from spreading, postmortem investigation is crucial for determining the causes of death, examining complications, spotting lung injury patterns, and assessing the virus’s population spread [11].

This article’s goal is to highlight the imaging, macroscopic, and histopathological aspects of viral pneumonia and multi-resistant bacterial pneumonia by focusing on patients with traumatic injuries, because these patients are prone to developing nosocomial pulmonary infections due to prolonged immobilization and invasive maneuvers to which they are subjected.

Even if the SARS-CoV-2 pandemic has passed, isolated Coronavirus infections still exist. Our study emphasizes the necessity to distinguish the thanatogenetic role of a viral infection from a bacterial nosocomial infection, to be able to establish an appropriate diagnosis and management of a case in forensic practice.

## 2. Materials and Methods

This study is retrospective in nature and is based on forensic and medical records.

We selected the cases of patients that were autopsied at the Institute of Forensic Medicine Timisoara between 1 June 2020 and 31 December 2021 that met with three inclusion criteria:

- Suffered a traumatic injury that led to hospitalization;
- Had a confirmed SARS-CoV-2 infection either at the beginning or during hospitalization;
- Had nosocomial bacterial bronchopneumonia which was either diagnosed clinically or postmortem.

Exclusion criteria: medico-legal autopsy records of people that were not victims of traumatic injuries, that did not have a SARS-CoV-2 infection or nosocomial bacterial bronchopneumonia, and records not pertaining to the period mentioned in the inclusion criteria above were excluded.

From 1 June 2020 to 31 December 2021, 1048 autopsies were performed at the Institute of Forensic Medicine Timisoara. From all these cases, 189 (18.03%) were patients with traumatic injuries hospitalized for more than 48 h, and 110 of them (10.49%) had a clinical or postmortem diagnosis for a nosocomial infection. From all these 110 cases, 9 had a confirmed SARS-CoV-2 infection either at the beginning or during hospitalization.

We studied the autopsy reports and the medical documentation of the patients.

The parameters we analyzed were: age of the patients, the hospitalization period, the type of traumatic injuries they suffered, any pre-existing illnesses before hospitalization, if the patient has undergone surgery, if the patient was intubated, pulmonary imaging investigations, SARS-CoV-2 test results, the clinical diagnosis of SARS-CoV-2 pneumonia, the clinical diagnosis of nosocomial bronchopneumonia, the result of bronchial aspirate cultures, the macroscopic aspects of the lungs at autopsy, the histopathological examinations of lung tissue, and the cause of death.

We are obliged to mention some limitations of the study, including the small number of cases, the summary medical documentation, and the lack of microbiological tests during hospitalization in most cases.

## 3. Results

After applying the inclusion criteria, we obtained nine cases that fulfilled all the three requirements.

All the cases were hospitalized at Emergency County Hospital “Pius Brânzeu” Timişoara (ECHPBT), and after the patients died, they were transferred to the Institute of Forensic Medicine Timisoara for medico-legal autopsy.

In Table 1 we illustrate the cases with the parameters we analyzed (age of the patient, hospitalization unit and period, traumatic injuries, pre-existing illnesses, if surgery or intubation was performed).

From the available medical documents, it appears that only two cases were vaccinated against SARS-CoV-2 (case 4 and case 8). For the other cases we had no information regarding the vaccination status. Moreover, we had no data regarding a previous SARS-CoV-2 infection of the patients.

Next, we summarize the relevant changes in the patients’ evolutions per days in relation to paraclinical investigations during hospitalization (SARS-CoV-2 test, chest-CT, microbiology test):

Case 1:

Day 0: negative SARS-CoV-2 test result
Day 0: discrete interstitial reticular infiltration
Day 6: process of consolidation associated to air bronchograms of the left lower lobe of the lung shown on computed tomography (CT) scan.
Day 8: bilateral basal pneumonia.
Day 13: positive SARS-CoV-2 test result.
Day 16: bronchial aspirate culture—*Staphylococcus aureus* (*S. aureus*).

Case 2:

Day 0: negative SARS-CoV-2 test result
Day 0: consolidation pulmonary areas with a pyramidal shape and intrapleural fluid accumulation (CT)
Day 5: bronchial aspirate culture—*Enterobacter aerogenes*.
Day 11: bronchial aspirate culture—*Acinetobacter baumannii* (*A. baumannii*) multi-resistant.
Day 13: bronchial aspirate culture—*A. baumannii* multi-resistant, *Providencia stuartii*.
Day 18: bronchial aspirate culture—*A. baumannii* multi-resistant.
Day 22: focus of consolidation in the upper right lung lobe (CT).
Day 25: bronchial aspirate culture—*Pseudomonas aeruginosa* (*P. aeruginosa*) multi-resistant.
Day 35: bronchial aspirate culture—*Proteus mirabilis*.
Day 36: multiple areas of pulmonary consolidation, pleurisy (CT).
Day 37: bronchial aspirate culture—*A. baumannii* multi-resistant, *Proteus mirabilis* multi-resistant.
Day 46: bronchial aspirate culture—*P. aeruginosa* multi-resistant.
Day 71: bronchial aspirate culture—*Pseudomonas spp.*
Day 82: bronchial aspirate culture—*P. aeruginosa* multi-resistant.
Day 95: positive SARS-CoV-2 test result.

Case 3:

Day 0: positive SARS-CoV-2 test result.

Case 4:

Day 0: positive SARS-CoV-2 test result.
Day 0: multiple areas of pulmonary consolidation CO-RADS (COVID-19 Reporting and Data System) 2 (CT).

Case 5:

Day 0: positive SARS-CoV-2 test result.
Day 0: multiple areas of pulmonary consolidation CO-RADS 3, fibronodular and calcareous pulmonary lesions (CT).

Case 6:

Day 0: positive SARS-CoV-2 test result.
Day 10: viral pneumonia with 40% lung damage (CT).

Case 7:

Day 0: positive SARS-CoV-2 test result.
Day 0: normal lung appearance (CT).
Day 3: “ground glass” lesions and small consolidation areas (CT) on the 15% of the lung volume.

Case 8:

Day 0: positive SARS-CoV-2 test result.
Day 0: inhomogeneous veiling of the lung fields, accentuated interstitial pattern, diffuse alveolar infiltrates (X-ray).
Day 2: “ground glass” diffuse lesions and bilateral basal consolidation areas.
Day 12: the extension of consolidation areas bilateral.

Case 9:

Day 0: negative SARS-CoV-2 test result
Day 1: small consolidation areas, accentuated interstitial pattern, minimal pleurisy (CT).
Day 9: resorption of condensation areas (CT).
Day 13: positive SARS-CoV-2 test result.
Day 19: condensation areas (CT) on the 15% of the lung surface.
Day 26: alveolar opacities—CO-RADS 5 lesions on the 80% of the lung volume.

Analyzing the investigations performed to patients at admission and during hospitalization we noticed that:

- SARS-CoV-2 tests and CT scans or X-rays and bronchial aspirate cultures were performed for 22.22% of patients (*n* = 2);
- SARS-CoV-2 tests and CT scans or X-rays were performed for 66.66% of patients (*n* = 6);
- only SARS-CoV-2 tests were performed for 11.11% of patients (*n* = 1).

When they were admitted to the hospital, all of the patients had undergone SARS-CoV-2 testing. Therefore, their treatment management was different according to the test results; if it was positive, the patients were redirected to specially arranged spaces (COVID wings). In our study, 66.66% (*n* = 6) of the patients were positive at admission and 50% of them needed surgery (*n* = 3) for the traumatic injuries they presented.

In 66.66% of patients (*n* = 6), pulmonary imaging investigations were performed at admission (CT scan or X-ray). Pulmonary imaging studies in patients who had a positive SARS-CoV-2 test result at admission revealed areas of pulmonary consolidation located predominantly basally (two or three CO-RADS lesions) in three cases (50%) and interstitial thickening associated with air bronchograms (one case). The other patients presented a normal imaging investigation at admission. In the evolution of all patients with areas of pulmonary consolidation, we noticed the appearance of “ground glass” opacities (GGO), as well as new (and increasing) consolidation areas in the existing ones after a variable period.

Three cases that had a negative SARS-CoV-2 test result at admission presented another type of CT pulmonary lesions, such as pulmonary contusions described as consolidation areas with a pyramidal shape and intrapleural fluid accumulation (traumatic injuries) (Case 2), discrete interstitial reticular infiltration (Case 1), and small consolidation areas, accentuated interstitial pattern, and minimal pleurisy (Case 9).

In Case 1, the pulmonary CT examination performed at admission showed reticular interstitial infiltration, the bronchial aspirate culture was positive for *S. aureus* after 6 days of hospitalization, and CT examinations showed left lower lung lobe consolidation (lobar pneumonia) and presence of air bronchograms. After another 6 days, the SARS-CoV-2 test result became positive; no more CT examinations were performed.

In Case 2, repeated bronchial aspirate cultures were performed, which all came out positive for multi-resistant bacteria (*A. baumannii*, *P. aeruginosa*, *Providencia stuartii*, and *Proteus mirabilis*) 11 days after admission. In evolution, CT examination showed lung consolidation areas, unorganized opacities, presence of air bronchograms, and bilateral pleurisy. After the result of the SARS-CoV-2 test became positive on the 95th day from admission, no other CT examination was performed.

In Case 3, only a SARS-CoV-2 test was performed at admission.

In Cases 4 and 5, CT scans performed on the admission day showed multiple areas of pulmonary consolidation. The SARS-CoV-2 test result was positive. Microbiological examinations were not performed during hospitalization.

In Case 6, a CT scan performed on the 10th day after admission showed elements of viral pneumonia with a 40% lung damage. We had no data regarding a bacterial pneumonia.

In Case 7, the SARS-CoV-2 test result was positive at admission without imaging features suggestive of pneumonia.

In Case 8, a positive SARS-CoV-2 test result on admission and inhomogeneous veiling of the lung fields, accentuated interstitial pattern, and diffuse alveolar infiltrates on chest X-ray were noted.

In Case 9, the CT performed the next day after admission showed minimal pleurisy, small localized basal bilateral consolidated pulmonary areas, and moderate interstitial thickening.

One case had a pre-existing pulmonary pathology (sequelae after tuberculosis) which can represent a risk factor for a viral or bacterial pneumonia. The other seven cases also had pre-existing illnesses which could influence the evolution of the cases in a negative manner. In Case 9, a CT scan performed the next day after admission showed small consolidation areas, an accentuated interstitial pattern, minimal pleurisy, and evolution to resorption (day 9), which can suggest traumatic injuries or a pre-existing pneumonia.

We should mention also that 55.55% (*n* = 5) of the patients were intubated, which represents a high risk factor for nosocomial pneumonia.

After the death of the patients, a medico-legal autopsy was mandatory, ordered by the police, due to the traumatic injuries the patients presented. The autopsies of the patients revealed changes in the macroscopic aspects of the lungs and histopathological findings on lung tissues.

**Figure 1:**
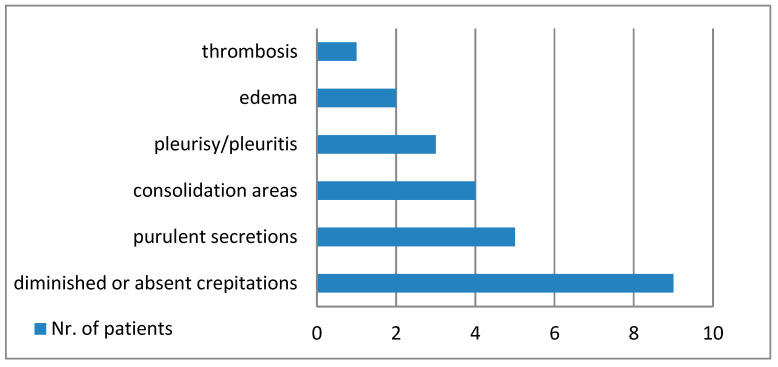
Macroscopic changes of the lungs.

**Figure 2:**
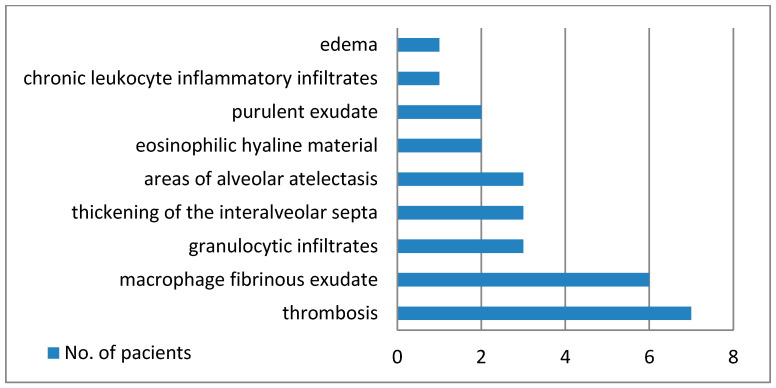
Histopathological pulmonary findings.

Figure 1 shows the main macroscopic findings of the lungs, and Figure 2 establishes the microscopic modifications that were discovered.

Out of all of the patients, two cases had clinical diagnosis for nosocomial bacterial bronchopneumonia before being SARS-CoV-2 positive. The other seven cases presented pulmonary findings suggestive for bacterial bronchopneumonia at autopsy and seen on histopathological examination (purulent secretion in lower bronchi, areas of serous alveolitis, serohematous alveolitis, granulocytic alveolitis, fibrino-leukocyte exudate, and pus in the lumen of the bronchioles).

The macroscopic pulmonary changes seen during the autopsy of the nine cases were varied, but we discovered suggestive elements for a definite etiological diagnosis. Macroscopic purulent secretions in the lower respiratory tract were a marker for bacterial infection, associated with lung consolidation and pleurisy.

**Figure 3:**
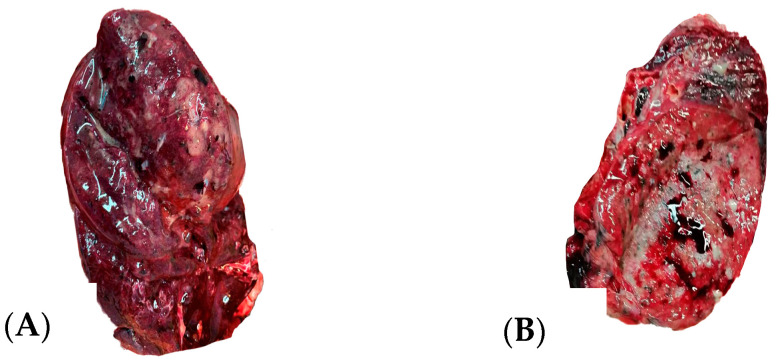
Sectioned lung at autopsy: macroscopic aspect of bronchopneumonia: consolidation of the lung with edema and thrombus (**A**), condensed lung, purulent secretion in lower bronchi (**B**).

Figure 3 illustrates the macroscopic findings of a sectioned lung at autopsy.

The medico-legal autopsy was completed with histopathological examination of lung tissues harvested at autopsy, that showed numerous and polymorphous findings:

- thrombi in seven cases;
- areas of serous alveolitis, serohematous alveolitis, granulocytic alveolitis at five cases;
- macrophage and fibrinous exudate in five cases;
- thickening of interalveolar septa in three cases;
- areas of alveolar atelectasis in three cases;
- fibrino-leukocyte exudate and pus in the lumen of the bronchioles in two cases;
- deposits of eosinophilic hyaline material in two cases;
- hematous microfoci in one case;
- edema in one case;
- acute emphysema in one case.

Paraffin was used to implant lung tissue and 3 μm slides were cut and stained with hematoxylin and eosin.

**Figure 4:**
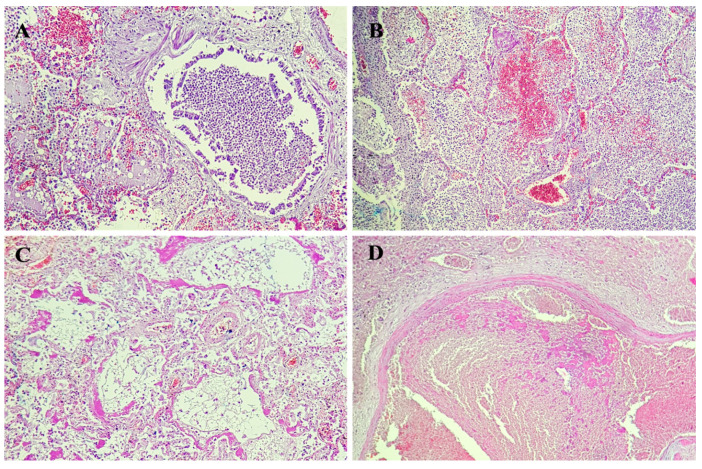
Lung tissue with bronchiole with purulent exudate (**A**), condensation area with different types of alveolitis (granulocytic alveolitis, fibrinous and leucocytic alveolitis, hemorrhagic alveolitis) (**B**), hyaline membranes on alveolar duct or sacs (**C**), and intraluminal thrombus (**D**).

Figure 4 illustrates some of the microscopic findings on lung tissue.

## 4. Discussion

The entire acute trauma care chain has been impacted by the COVID-19 pandemic’s organizational changes and shortage of medical resources, which may have an effect on patient outcomes [12].

Hospitalized patients’ compromised immune systems and lung injury from COVID-19 can create an ideal setting for the growth and colonization of microorganisms. Mechanical ventilation is blamed for secondary bacterial and fungal infections that co-occur with viral respiratory infections [7].

All the patients from our study were tested for SARS-CoV-2 infection at admission in the medical unit care, and for 66.66% of them, thoracic CT or chest X-rays were performed. Six out of nine patients were positive for SARS-CoV-2 at admission.

Despite national and international recommendations, the use of CT has expanded dramatically during the COVID-19 epidemic [13]. The majority of the research indicates that bilateral subpleural GGOs with lower lobes and posterior predominance is the typical CT appearance of SARS-CoV-2 lung involvement. Lung consolidations, bronchiectasis, nodules, pleural thickening, interstitial thickening, a crazy-paving look, halo sign, and linear opacities were less common [14,15]. We considered GGOs with or without consolidation or visible intralobular lines as a typical CT finding for COVID-19 pneumonia [7].

COVID-19 pneumonia is characterized by alveolar interstitial damage with inflammatory exudation/edema, and a CT scan can provide a differential diagnosis and assist in assessing the severity of lung disease and the existence of complications [13]. Air bronchograms and poorly defined patchy infiltrates dispersed throughout the lungs are suggestive markers for bronchopneumonia [15].

In our study, two out of six patients which were positive for SARS-CoV-2 at admission presented typical changes for SARS-CoV-2-pneumonia on CT investigation: small localized basal bilateral consolidation lung areas—CO-RADS 2 score, diffuse GGOs. In their evolution, we noticed the extension of pulmonary consolidation areas despite the treatment.

Seven of the twelve patients in the first study on COVID-19 autopsy had deep vein thrombosis, and four of them died from pulmonary embolism. The most frequent histological findings in the lungs were interstitial edema, activated pneumocytes, hyaline membranes, diffuse alveolar damage (DAD), and microvascular thrombosis. The studies that followed confirmed these aspects for the description of SARS-CoV-2 pneumonia. Alveolar damage, encompassing the exudative and proliferative phases, is the predominant type of lung injury in SARS-CoV-2 pneumonia. Numerous cases included fibrinous exudate, edema, pneumocyte hyperplasia, inflammatory infiltration, and organization [16].

The acute phase of DAD is demonstrated by the majority of pathologic findings in postmortem lung biopsies of seven deceased SARS-CoV-2 infected patients in our investigation. Alveolar wall edema, fibrinoid exudate, and the development of hyaline membranes are all linked to the acute phase of DAD. Proliferative and fibrotic phases, more severe DAD (diffuse alveolar damage) phases, have been documented in literature as well [17].

Vascular alterations associated with proliferative and exudative diffuse alveolar damage with hyaline membrane deposits, necrosis of alveolar lining cells, TTF-1 positive type II pneumocyte hyperplasia with nucleomegaly, and prominent nucleoli alternatively combined with the accumulation of lymphocytes, macrophages, and multinucleated giant cells were regarded as distinguishing characteristics of COVID-19 infection [18].

In particular, all disease stages had epithelial alterations, such as reactive pneumocyte atypia, denudation, and DAD; additionally, vascular changes such microvascular damage, thrombi, intra-alveolar fibrin deposits, and other traits of acute fibrinous and organizing pneumonia were also present in the early stages of symptomatic COVID-19 infection [19].

Pulmonary infarcts might also occur in intubation in ARDS (acute respiratory distress syndrome). These results imply that pulmonary microthrombi are linked to ARDS but may not be unique to COVID-19 [20].

The clinical diagnosis of nosocomial bronchopneumonia was established according to the order of the Ministry of Health of Romania no. 1106/2016 and decision 2012/506/UE, based on the following: imagistic criteria, symptomatology and clinical examination, and microbiology tests. The severity of a patient’s underlying illnesses may be a risk factor for the development of HAP; for instance, coma and head trauma injuries play an important role in the selection and colonization by microorganisms, such as *Staphylococcus aureus* [21]. In our study, only two cases had a microbiological diagnosis for nosocomial bronchopneumonia, one case a with *Staphylococcus aureus* positive test and the other with an association of multi-resistant germs (*Acinetobacter baumannii*, *Pseudomonas aeruginosa*, *Proteus mirabilis*, and *Enterobacter aerogenes*).

In most cases, the etiology of nosocomial bronchopneumonia is bacterial; however, according to a recent systematic review, the hospital-acquired severe acute respiratory syndrome coronavirus 2 (SARS-CoV-2) infection rate has become 12–15% [22]. One of the issues in relation to COVID-19 is obtaining data that differentiate between healthcare versus community sources of bacterial infection in patients. Early research is showing that COVID-19 patients have a small but considerable chance of contracting an HAI after being admitted to the hospital. The severity of COVID-19 illness and the length of hospital stay significantly raise this risk [23]. The possibility of co-infection with other microbial pathogens should be considered in patients with COVID-19, especially in elderly patients, given the severe and potentially fatal consequences of bacterial infections. This is highly important due to the difficulty in identifying bacterial co-infection on computed tomography images alone, after the development of acute respiratory distress syndrome.

Although some authors have acknowledged that the number of nosocomial infections has dropped during the pandemic, maybe as a result of increased personal protective equipment and adjusted procedures (earlier discharge of stable trauma patients and restricted visiting hours) [24], our opinion is that patients with bacterial pulmonary infections were underdiagnosed, microbiology tests were not performed, and their care was greatly influenced by the COVID-19 infection.

The radiological findings in COVID-19 are indicative of DAD, vascular damage, and thrombosis, or a combination of both. However, there is no correlation between the several usual CT patterns in COVID-19 and particular histological findings [25].

In Cases 3, 4, 5, 6, 7, and 8, the nosocomial bacterial bronchopneumonia occurred after the SARS-CoV-2 pneumonia, most likely favored by the lung damage caused by the virus.

In Case 1, we cannot establish which infection occurred first, the bacterial or the viral infection, and both meet the criteria of a nosocomial infection.

In Case 2, we are dealing with repeated pluri-bacterial infections during hospitalization and a positive SARS-CoV-2 test on the 95th day of hospitalization, all nosocomial infections.

In Case 9, considering the lack of microbiological tests during hospitalization, we cannot establish when the bacterial infection occurred, but based on imaging investigations it is clear that this is a super-infection after SARS-CoV-2 infection.

The coinfecting pathogens can be detected at different times during the COVID-19 disease course and continue to be a crucial factor in targeted treatment strategies for COVID-19 patients [26].

Lastly, it is critical to take into account how the several significant comorbidities (77.77% of all discussed cases, *n* = 7) contribute to the dying process. The small number of cases did not allow us to make suppositions regarding the negative impact of these illnesses in the unfavorable evolution of the patients and to establish their contribution to death. Stated differently, are all of the postmortem results associated with the COVID-19 infection? Since the question remains unanswered, it would be preferable to elucidate this in subsequent research [18].

Since the evaluation occurs at the conclusion of the disease course, postmortem studies are prone to constraints, the most significant of which is the inability to analyze sickness dynamics. For this reason, we should address the study’s limitations [27]. Our study is limited due to the small sample size and population distribution, and also by the impossibility to perform postmortem microbiological tests in our institute, which could represent an objective proof of a nosocomial infection.

However, we think that autopsies in COVID-19 infection cases are a crucial part of the peer review procedure, with a careful comparison of pathological and clinical data [28].

## 5. Conclusions

By offering vital information on diagnosis, cause of death assessment, and death management, forensic pathology was essential in the control of the COVID-19 epidemic.

Our study emphasizes the fact that during the hospitalization of patients with traumatic injuries, they can develop both viral and bacterial pneumonia. When performing the autopsy of these cases, we can differentiate between the two entities by studying the imaging aspects of lung tissues, both macroscopic and microscopic. Determining the cause of death and the function that each pathology plays in the thanatogenerative process can be greatly aided by this separation.

We think that the aspects we highlighted in this study, regarding the macroscopic and microscopic findings in lung tissue, although on a small sample, can be very useful in forensic practice in cases with a pluri-factorial pathology.

## Figures and Tables

**Table 1:** Clinical features of selected patients.

No. of Case	Age	Hospitalization Unit and Period	Traumatic Injuries	Pre-Existing Ilnesses	Surgery	Intubation
Case 1	79	ECHPBT—Neurosurgery 17 days	Craniocerebral trauma	Cardiovascular diseases	Yes	Yes
Case 2	55	ECHPBT—Neurosurgery 110 days	Spine and thoracic trauma	Chronic alcoholism	Yes	Yes
Case 3	82	ECHPBT—Neurosurgery 5 days	Craniocerebral Trauma	Pneumonia, Cardiovascular diseases	No	No
Case 4	44	ECHPBT—Burnts unit 6 days	Burn injuries	Obesity	Yes	Yes
Case 5	56	SCHPBT—Orthopedic 3 days	Femure fracture	Sequelae after pulmonary tuberculosis	No	No
Case 6	69	ECHPBT—Burnts unit 14 days	Burn injuries	Asthmatic bronchitis, Obesity, Cardiovascular diseases	Yes	No
Case 7	81	ECHPBT—Neurosurgery 10 days	Craniocerebral trauma	Cardiovascular diseases	No	No
Case 8	73	ECHPBT—Neurosurgery 14 days	Craniocerebral trauma	No	Yes	Yes
Case 9	68	ECHPBT—Neurosurgery 28 days	Craniocerebral trauma	Cardiovascular diseases	No	Yes

## Data Availability

The original contributions presented in the study are included in the article, and further inquiries can be directed to the corresponding author.
